# Differential Molecular Transcriptomics and Underlying Biological Pathways between Smokers Lung Cancer (Small Cell Lung Carcinoma) in Comparison to Healthy Human Lung Cells

**DOI:** 10.4236/cmb.2025.152002

**Published:** 2025-06-26

**Authors:** Brent Lake, Anasua Banerjee, Jazmine Cuffee, Narendra Banerjee, Erik Armstrong, Satyendra Banerjee, Coza Blount, Colby Hunter, Ahmed El-Hashash, Kuldeep Rawat, Dolapo Adedeji, Somiranjan Ghosh, Tanmoy Mondal, Zahidur Abedin, Hirendra Nath Banerjee

**Affiliations:** 1 Department of Natural Sciences, Pharmaceutical Science, Computer Science, Engineering & Technology, Elizabeth City State University Campus of the University of North Carolina, Elizabeth City, USA; 2 Department of Biology, Howard University, Washington, USA; 3 Departments of Pediatrics and Child Health, College of Medicine, Howard University, Washington, USA; 4 PrimBio Research Institute, Garnet Valley, USA

**Keywords:** Lung Cancer, Transcriptome, Core Canonical Pathways

## Abstract

Cancer is a public health crisis affecting approximately 19.3 million globally annually. Lung cancer has the highest percentage of incidence and is responsible for 27% of cancer-related deaths. The lung cancer of prevalence in smokers is small-cell lung carcinoma. Lung cancer cells often exhibit altered gene expression patterns, compared to their normal counterparts. Yet this differential gene expression is not well understood. Herein, we investigated the differential expression of genes in small cell cancer cell lines compared to a healthy lung cell line that can lead to further insights into the proliferation, metastasis, and drug resistance of these lung cancer cells. Whole transcriptomic and small non-coding RNA sequencing was done by Next-generation sequencing (NGS) method, coupled with the Ingenuity Pathway Analysis (IPA) to decipher the core canonical pathways involved in these lung cancer cell-signaling mechanisms in disease pathophysiology. The findings of this investigation identified multiple genes and small non-coding tRNA/tRNA fragments that were differentially expressed, which thereby showed the possibility of potential diagnostic/prognostic and therapeutic targets. This important transcriptomic analysis can be a valuable tool for developing more effective treatment strategies for smokers lung cancer.

## 1. Introduction

Cancer is a disease in which a group of normal cells disrupts the homeostatic state of the body and transforms into malignant metastatic cells. Almost every tissue of the body may be affected by cancer, and some may produce several types. The earliest recorded information about cancer can be found in an Egyptian papyrus dating back to around 3000 BC [1]. The term cancer comes from the Greek word “Carcinos” and carcinoma “Kankram” (Hippocrates, 460–370 BC). The term metastasis is derived from the Greek word “Methistemi” meaning to remove or set free [2]. Hanahan and Weinberg [3]. have explained the hallmarks of cancer as acquired functional capabilities that allow cancer cells to survive and proliferate. These hallmarks of cancer constitute sustaining proliferative signaling, evading growth suppressors, resisting cell death, enabling replicative immortality, inducing angiogenesis, activating metastasis, altering cellular metabolism, and avoiding immune destruction.

Lung cancer is among the most common tumor types, representing 13% of newly diagnosed cancers worldwide. The prevalence of lung cancer has risen dramatically as the leading cause of cancer-related deaths, accounting for 18% of the total number of deaths [4]. Approximately 20% of lung tumors exhibit neuroendocrine differentiation, representing a group of neoplasms that share common morphological and immunohistochemical features, including small cell lung cancer (SCLC). SCLC, accounting for 10% of clinical lung cancer cases, is an aggressive malignancy strongly associated with smoking. It demonstrates a distinct natural history characterized by a high growth fraction, rapid doubling time and early establishment of widespread metastatic lesions. While 30% of patients present with disease confined to one hemithorax [limited disease (LD)], most cases have disease not encompassed by one radiotherapy field [extended disease (ED)]. Despite a higher prevalence of smoking in African American men and women, the SCLC is less prevalent in African American individuals than in white Americans. SCLC tumor mutational profiling reveals a clear smoking signature, providing direct evidence that tobacco carcinogens are responsible for the initiation of SCLC, Concomitant inactivation of two tumor suppressors, i.e., p53 and RB (encoded by TP53 and RB1, respectively) is found in most SCLC cases. This dual inactivation of tumor suppressors is distinct from the primary oncogenic drivers of many other solid tumors, notably non-small-cell lung cancers (NSCLC), in which activating oncogenic mutations seem to be essential for tumorigenesis. Changes in the lung stroma and immune microenvironment also presumably contribute to SCLC tumorigenesis. Moreover, the loss of p53 and RB1 occurs frequently in SCLC. Other studies described the amplification of MYC family genes (MYC, MYCL and MYCN) in a subset of SCLC tumors. Among the few that have been functionally validated in mouse models or cell culture assays are loss-of-function events in RB family members p107 and p130 (encoded by RBL1 and RBL2, respectively), the tumor suppressor PTEN, NOTCH receptors and the chromatin regulator CREBBP. In addition to recurrent amplification of MYC family genes, amplification of FGFR1 (encoding fibroblast growth factor receptor 1) and GNAS (encoding the α-subunit of the heterotrimeric G protein Gs) also occurs. The histone methyltransferase KMT2D (also known as MLL) is mutationally inactivated in 8% of SCLC tumors.

Both RB and p53 play key roles in regulating cell cycle progression. RB is a major inhibitor of S phase entry, whereas p53 is integral to multiple cell cycle checkpoints, triggering cell cycle arrest or inducing apoptosis in response to various cellular stresses. The loss of p107 or p130, amplification of MYC family members, alterations in the PTEN pathway, and high expression of BCL-2 have all been implicated in promoting cell growth, proliferation, and survival in SCLC. The abrogation of the G1-S cell cycle checkpoint associated with the loss of p53 and RB results in an increased reliance on subsequent cell cycle checkpoints to ensure genome stability and correct chromosomal segregation. Accordingly, the inhibition of kinases that are important for the G2-M transition, such as ATR, WEE1 and CHK1, promotes mitotic catastrophe in SCLC cells, and these kinases are being explored as therapeutic targets. Similarly, the dysregulated cell cycle progression in SCLC and the resulting DNA damage may render SCLC vulnerable to multiple strategies that inhibit DNA repair pathways. The activation of the PI3K-AKT-mTOR pathway has been implicated in proliferation and resistance to apoptosis in SCLC.

In this study, we investigated the differential gene expression in a small cell lung cancer cell line in comparison to a healthy lung cell line analyzing the transcriptomic data by the Ingenuity Software System (IPA^®^) to decipher the cellular signaling mechanism and the core canonical pathways (CP) involved in the dreaded lung cancer cells, causing the highest rate of mortality in smokers all over the world.

## 2. Methods and Materials

### 2.1. Cell Culture

Cancer cell line HTB-172 (ATCC-NC1-H209) from a 55-year-old Caucasian male was investigated in this research and was purchased from American Type Culture Collection (ATCC, Manassas, Virginia). In addition, the AG12211 (healthy lung cell line from a 54-year-old Caucasian male) was purchased from Coriell Institute for Medical Research (CIMS, Camden, NJ). Cell lines were cultured in aseptic conditions in a medium specific to each cell line (RPMI for HTB-172, and EMEM for AG12211) as per the culturing guidance of the ATCC and CIMS and maintained at 37degree Celsius in a CO_2_ incubator; Routine Mycoplasma detection were done to all cell lines using the MycoFlour Mycoplasma detection kit (Thermo Fisher Scientific, USA) using a Leica Fluorescence microscope.

### 2.2. Transcriptomic and Non-Coding RNA Sequencing

Total RNA was isolated from the AG12211 and HTB-172 cells by the standard Trizol isolation method and was sent for RNA sequencing through a servicer (PrimeBio, Exton, PA) to obtain transcriptomic and small non-coding RNA sequencing data. The servicer uses a pH-based Ion Torrent Platform, which uses a P1 chip and the Ion Proton sequencer. The transcriptomic information revealed the top five genes that are upregulated and downregulated according to the fold change and log fold change data, and these lead genes were assessed for their known function in cancer progression. In addition, the small non-coding RNA data were analyzed, and the top five most fold-changed (up and down) were assessed for their known function in cancer progression.

### 2.3. Ampliseq Transcriptome Methods

#### 1) Library Preparation:

cDNA libraries were constructed using Ion Ampliseq Transcriptome Human Gene Expression Kit from Thermofisher (Cat# A26325) and the manufacturers recommended protocol. Briefly, 100 ng of total RNA was reverse transcribed at 42°C for 30 minutes. After reverse transcription, the cDNA was amplified by PCR using Ion Ampliseq Transcriptome Human Gene Expression Core Panel primers that amplified the specific targets Step 1: 99°C for 2min; Step 2: 99°C for 15 sec, 60°C for 16 min for 16 cycles; Step 3: Hold at 10°C. Next, the primers were partially digested as directed. Following the partial digestion of primers, adapters and bar codes were ligated to the cDNA. The cDNA was then purified using AMPure XP reagent and recommended protocol. The purified cDNA libraries were then amplified by PCR using 1X Library Amp Mix and 2 μL of 25X Library Amp Primers with the conditions as follows: Step 1: 98°C for 2 mins; Step 2: 98°C for 15 sec, 64°C for 1 min; Steps 2–3 for 5 cycles and then hold at 10°C. The amplified cDNA libraries were purified using Nucleic Acid binding beads, binding buffers and run on Agilent 2100 Bioanalyzer to determine the yield and size distribution of each library.

#### 2) Bioanalyzer Library QC

The quality of each final library is assessed using the Agilent^®^ dsDNA High Sensitivity Kit. The molar concentration is determined from 50 – 1000 bp. The lower the percentage of DNA in the 50 – 160 bp range, the better the library is considered. Those with less than 50% of the library in this region are deemed ready for templating and sequencing.

#### 3) Templating, Enrichment and Sequencing

Approximately 100 pM of pooled barcoded libraries were used for templating using Thermofisher Ion PI^™^ Hi-Q^™^ OT2 200 Kit (Cat. # A26434)) and manufacturers recommended protocol. Briefly, 100 pM of pooled libraries were combined and 25 ul of each sample was loaded onto the Ion Chef. Next, all reagents for the Ion PI^™^ Hi-Q^™^ OT2 200 Kit were loaded onto the Ion Chef and the run was performed. The Ion Chef templates, enriches and loads the sample onto a P1 chip. After 15 hours the Chef pauses so that QC can be performed on the unenriched samples. After the pause the beads were isolated and quality assessment was performed on Qubit fluorometer to determine the % of beads that were polyclonal. After polyclonal assessment the Ion Chef resumed running and loaded the samples onto a P1 chip. The loaded chip was then placed into an Ion Proton sequencer, and the run was started using an Ion torrent Ampliseq transcriptome run plan that was configured based on type of library, species, number of run flows required, type of plug-in required, adapter-trimming as well as other parameters specific to the Ampliseq transcriptome run.

#### 4) Data Analysis

After completion of the proton run, the raw sequence files (fastq) were aligned to the human transcriptome (hg19) reference sequences by the StrandNGS software using the default parameters. The genes and transcripts that were used were retrieved from the Ensemble database. Aligned BAM files were used for further analysis. Quality control was assessed by the Strand NGS program, which determined the pre- and post-alignment quality of the reads for each sample. The aligned reads were then filtered based on read quality (≥ 15), alignment score (≥90), match count (≤1), mapping quality (≥25) and reads that failed vendors QC were removed. After filtering, the aligned reads were normalized and quantified using the DEseq algorithm by the StrandNGS program. Differentially expressed genes were then determined between each sample. After DEGs were identified, fold change was determined and genes that had a significant fold change of 2.0x or higher were listed.

### 2.4. Ingenuity Pathway Analysis (IPA)

Once the whole transcriptomic RNA sequencing data was determined, the data was processed using IPA software. Ingenuity Pathway Analysis (IPA).

Once the complete transcriptomic RNA sequencing data (DEG) had been generated, cellular processes and pathways were identified using Ingenuity Pathway Analysis (IPA, Ingenuity^®^ Systems, http://www.ingenuity.com), following the methodology as described by Qiagen, USA, the company that designs the software.. Briefly, gene identifiers and corresponding expression values (defined as differentially expressed with a fold change of ≥1.5 for upregulation or ≤−1.5 for downregulation) were imported into IPA. These differentially expressed genes were mapped to associated biofunctions, and interaction networks were algorithmically constructed based on gene connectivity.

The top-scoring network was identified by maximizing the number of connections among focus genes. Each network was labeled according to the predominant functional category represented. Canonical Pathway (CP) analysis was performed to identify function-specific genes significantly enriched within these networks.

To compare enriched pathways and upstream regulators across conditions, we used the comparison analysis feature in IPA. A heatmap was generated to visualize key canonical pathways and upstream regulators correlated with the experimental dataset. We used the IPA regulation z-score algorithm to identify biological functions that are expected to be more active in HTB-172 than AG12211 according to our RNAseq data. To enhance the stringency of our analysis. Activation z-scores were used to predict the activation state of biological functions, with scores ≥2 or ≤−2 considered significant, indicating increased or decreased activity, respectively.

## 3. Results

### 3.1. Total RNA Transcriptomic Data of AG12211 versus HTB-172

RNA sequencing data was used to determine differential gene expressions between small-cell lung cancer line HTB-172 and healthy lung cell line AG12211. The top three upregulated genes were GAS5-AS1, OR1A2, and EIF4A1P5 (Table 1).

The top three downregulated genes are EIF1B, NAPIL1, and TPT1 (Table 2). EIF1B has the greatest fold change of all the transcriptomic data, with a fold change of 97. Further investigation of the top 50 among the 372 genes in downregulation data revealed four known tumor suppressors. These tumor suppressor genes downregulated in cancer are PDCD4-AS1, RNF40, USP4, and ST13P5 (Table 3).

### 3.2. Small Non-Coding RNA Data of AG12211 versus HTB-172

The raw data mostly contained up or downregulation of transfer RNA (tRNA)/tRNA fragments. The tRNA/tRNA fragments that had an upregulation equal to or greater than 10-fold change were tRNA6, tRNA21, tRNA13, and tRNA14 (Table 4). The association/relationship to cancer of these tRNA/tRNA fragments is unknown.

The tRNA or tRNA fragments that had downregulation equal to or greater than 10-fold change were tRNA108, tRNA101, tRNA102 (Table 5). Only one tRNA has been found to be associated with cancer. tRNA101 mutations have been found to influence leukemia and lung cancer [5]. One miRNA (miR892B) was found in all the non-coding RNA sequencing data. miR892 regulation is involved in breast cancer progression [6].

### 3.3. Differential Gene Expressions in AG12211 Compared to HTB-172

**Figure 1.**
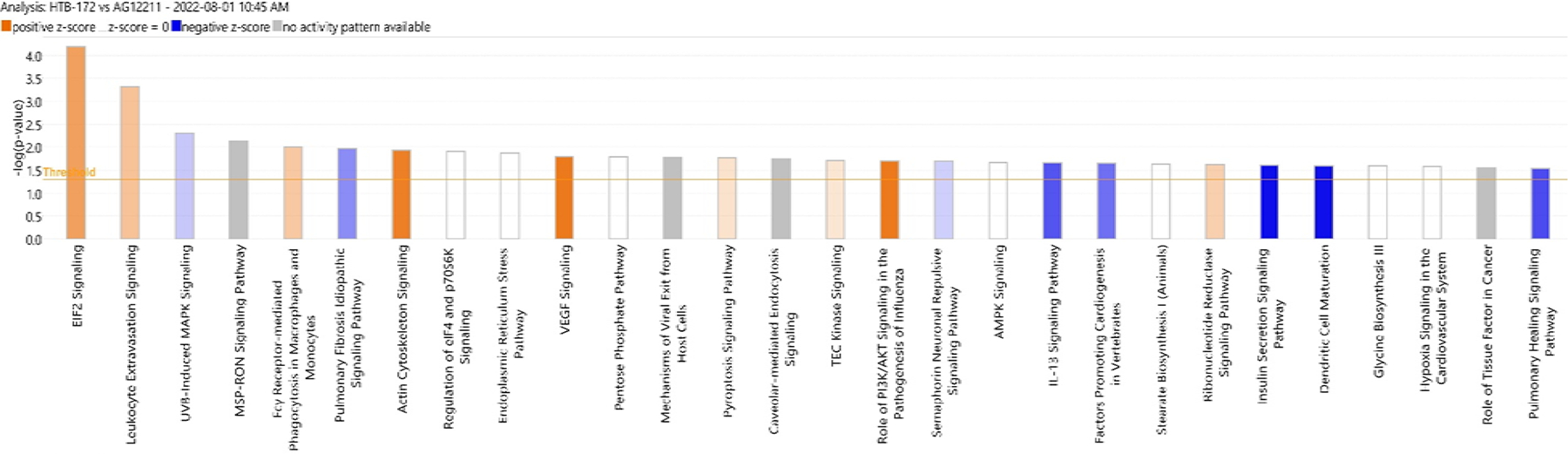
Core canonical pathways in Htb-172 versus Ag12211 computer modeled by IPA software analysis based on RNA sequencing data. The orange color represents predicted increase in expression and blue represents predicted decrease in expression.

The raw whole transcriptomic data of differential gene expression of small-cell lung cancer cell line HTB-172 compared to the healthy lung cell line AG12211 was processed by Ingenuity Pathway Analysis software. Based on the differential gene expression, IPA made a hierarchical model of upregulated and downregulated core canonical pathways involved in cancer progression and cell survival (Figure 1). These predicted core canonical pathways and their activation status (based on log fold change z-score) are shown in Figure 1. The orange color represents the upregulation of the pathway based on a positive z-score, and the blue color represents the downregulation of the pathway based on a negative z-score. The white color signifies that the activity is unknown, and the grey color signifies no activation in the pathway. The core canonical pathways of interest in this investigation are EIF2 signaling, VEGF signaling, IL-13 signaling, HOTAIR signaling, and UVB-induced MAPK signaling. The EIF2 signaling pathway has the highest positive z-score in the data.

**Figure 2.**
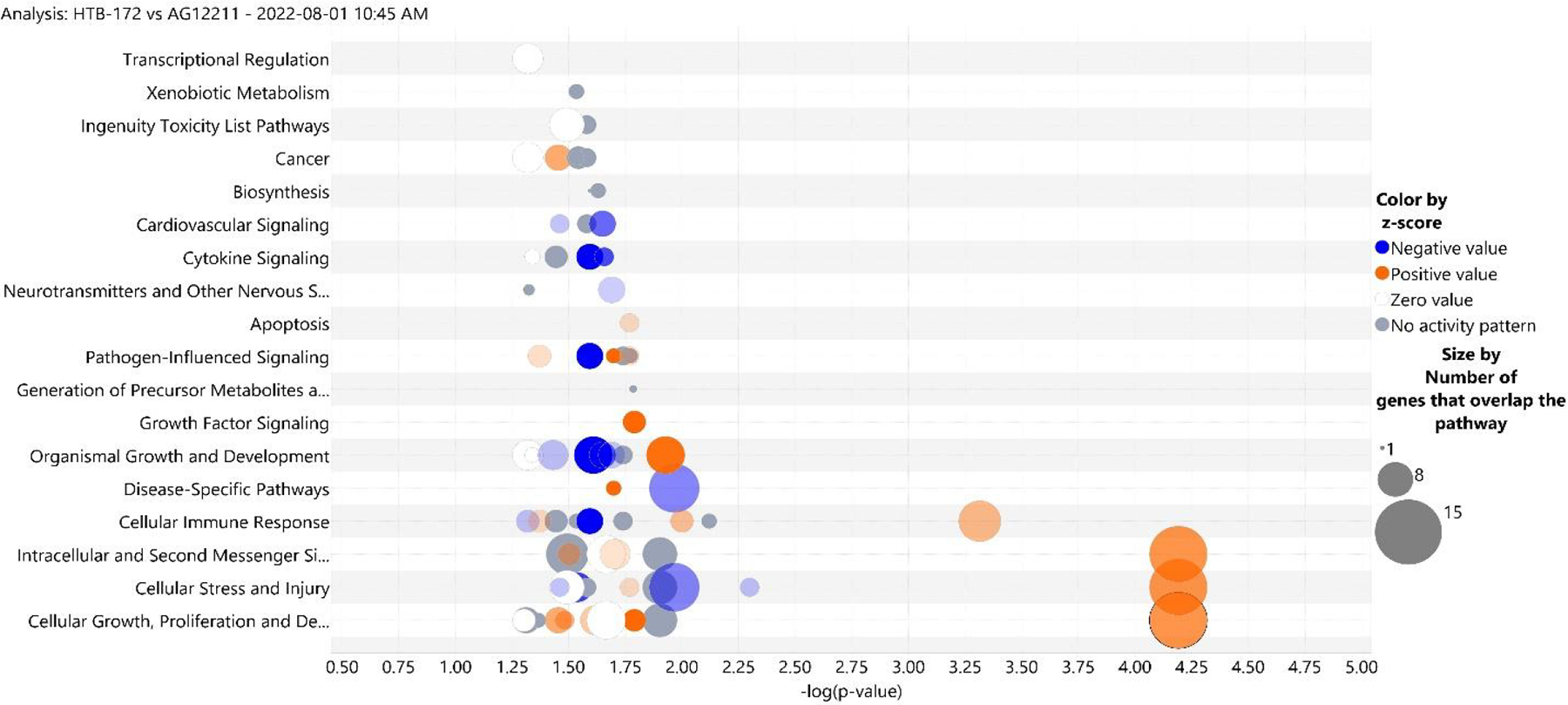
Predicted known canonical pathways gene regulation in AG12211 versus HTB-172 in the context of function and disease progression based on the log fold change activation score of the canonical pathways of HTB-172 versus AG12211. The color coding is the same as the canonical pathway bar graph, but the size of the circles corresponds to the number of genes involved in the pathway.

Figure 2 shows the predicted known canonical pathways of gene regulation in AG12211 versus HTB-172 in the context of function and disease progression. The color coding is the same as the canonical pathway bar graph (according to the log fold change activation z-score), but the size of the circles corresponds to the number of genes involved in the pathway. According to the chart, involved in cellular growth and proliferation, cellular stress and injury, and intracellular and secondary messenger signaling. As such, this pathway has the greatest number of genes involved.

**Figure 3.**
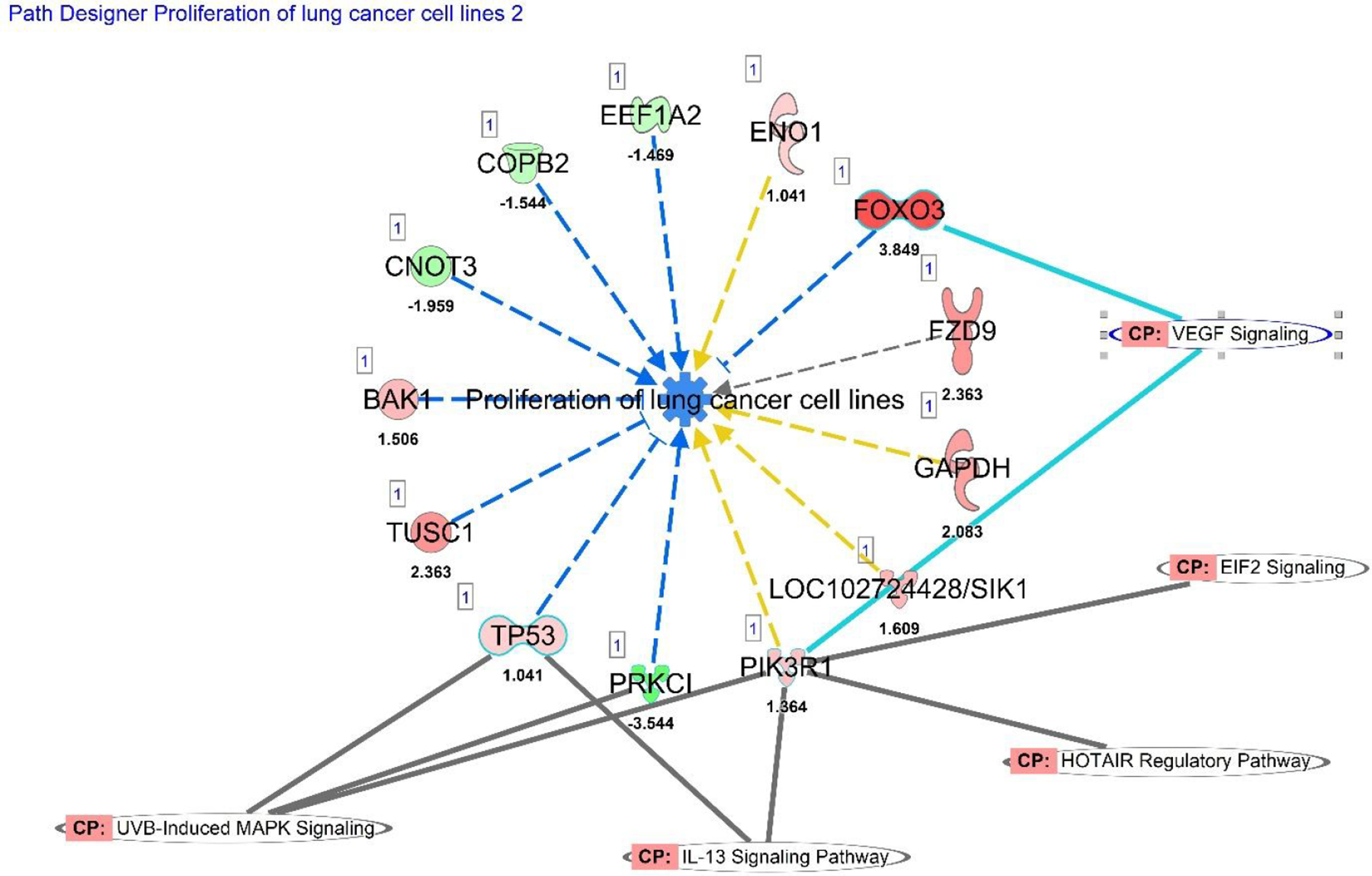
IPA Disease and Functions analysis of the RNA sequencing data for genes associated with proliferation of lung cancer cell lines. This pathway is overlayed with IL-13, UVB-Induced MAPK, HOTAIR Regulatory pathway, EIF2 and VEGF signaling canonical pathways. The expression data of each gene determines versus its known function determines whether it increases or decreases cell proliferation (Table 6 and Table 7). This pathway has an activation z-score of −1.103.

After the core canonical pathways were established, additional IPA analysis was done to investigate gene networks involved in the proliferation of lung cancer cell lines (Figure 3). In this model, the blue dashes with arrows represent indirect downregulation, and blue dashes with bracket ends represent indirect inhibition of cell proliferation. The yellow dashed lines represent indirect activation, and the grey dashed line indicates involvement but an unknown function. This category had an activation z-score of −1.103, which indicates predicted downregulation. Each of the upregulated and downregulated genes in this category were compared to their known functions, and IPA predicted effect based on the log fold change in Table 6 and Table 7.

Table 6 shows the oncogenes known to be involved in lung cancer cells proliferation, based on the upregulation log fold change of sequencing data. Based on the genes known involvement, the upregulation status in the raw data, these genes **are predicted to increase cell proliferation** Conversely, the genes in Table 7 are downregulated based on the RNA sequencing data, and all of these genes are known to be involved in cancer cell proliferation and Epithelial Mesenchymal Transition (EMT).

**Figure 4.**
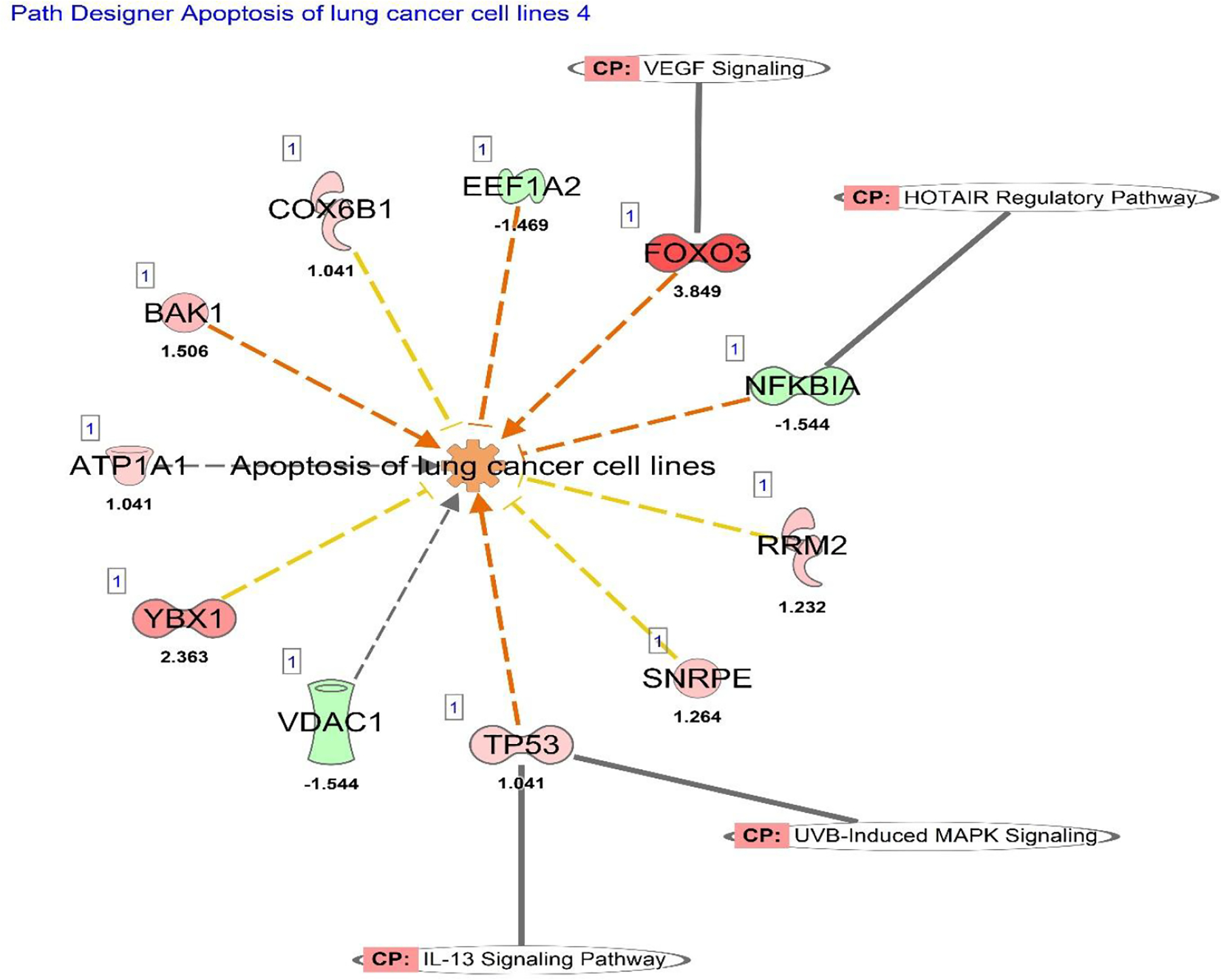
IPA Disease and Functions analysis of the RNA sequencing data for genes associated with apoptosis of lung cancer cell lines. This pathway is overlayed with IL-13, UVB-Induced MAPK, HOTAIR Regulatory pathway, and VEGF signaling canonical pathways. EIF2 signaling pathway did not have any associated genes. Each of these genes are known to be involved in the apoptosis of lung cancer cell lines. The expression data of each gene determines versus its known function determines whether it increases or decreases cell proliferation (Table 8 and Table 9). This pathway has an activation z-score of 0.862.

Analysis of the individual genes involved in apoptosis of lung cancer cell lines revealed the predicted interactions, as shown in Figure 4, the dashed yellow line indicates indirect activation, while the orange dashed line indicates indirect upregulation. The activation z-score of this data is 0.862, indicating this category’s upregulation.

Remarkably, the oncogenes shown are Known to decrease apoptosis that could result in cancer cell survival like YBX1, SNRPE, RRM2, and COX6B1 (Table 8).

**Figure 5.**
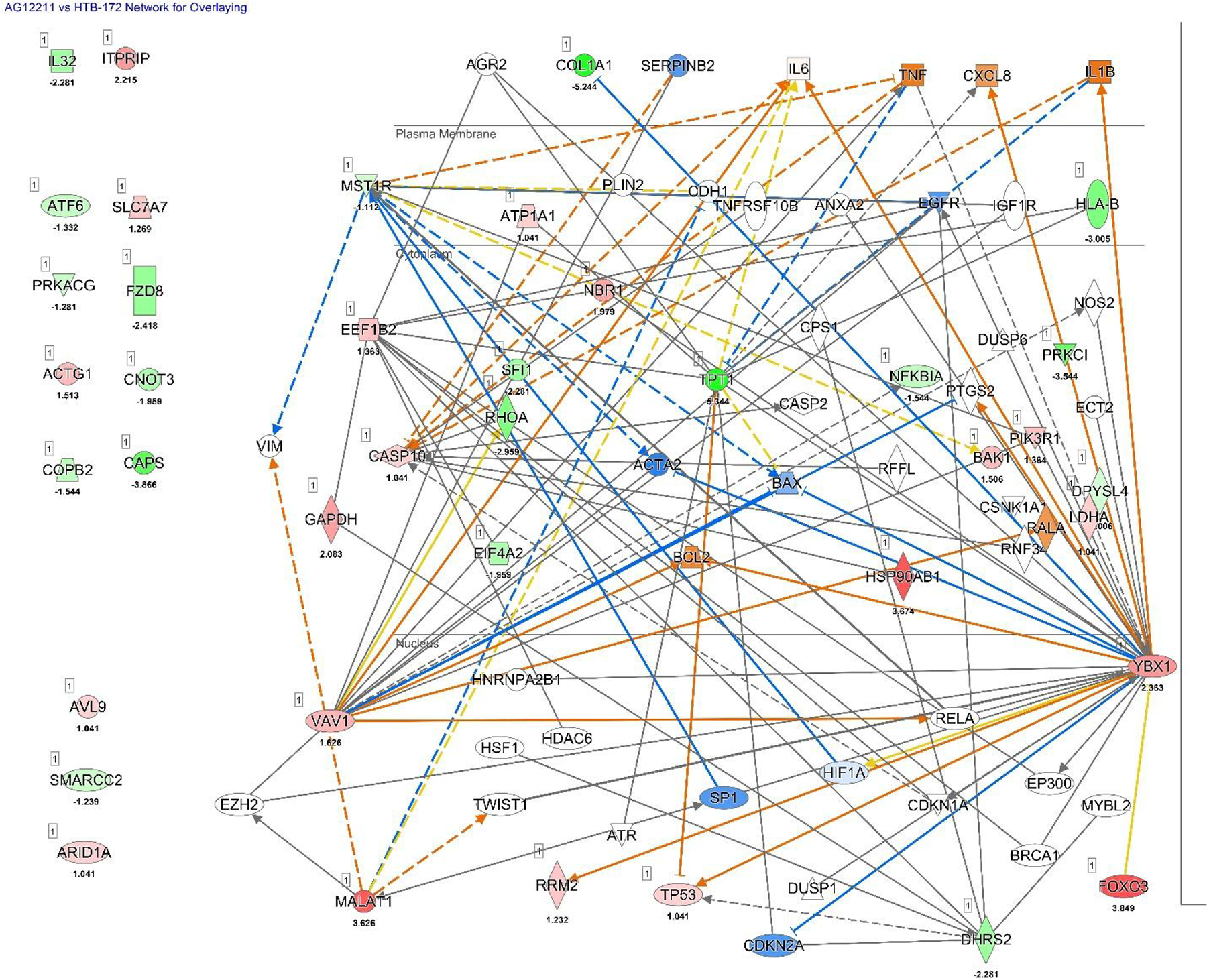
IPA analysis of AG12211 versus HTB-172. The top scoring network is associated with cellular movement, development, growth, and proliferation.

Next, we analyzed the genes associated with apoptosis, proliferation and the core canonical pathways from our data to determine the gene with the most associations between each pathway. As a base for this analysis, the top-scoring network predicted by IPA, which models interactions regarding cellular movement, development, growth, and proliferation, was used to overlay the established information (Figure 5). This pathway originally contained 142 genes, but the genes with no expression/data or genes that did not have any connections/relationships were trimmed down to 82 genes (Figure 5).

## 4. Discussion

In this study, the top three genes found to have the most significant fold change for upregulation and downregulation in AG12211 compared to HTB-172, were GAS5-AS1, ORLA2, and EIF4ALP5 genes that are oncogenic in various cancers, including lung cancer GAS5-AS1 has the greatest positive fold change in the data and is a long non-coding RNA established as an oncogene in lung and hepatocellular carcinoma [7]. OR1A2 is a g-protein protein-coupled receptor that is associated with olfactory recognition. Finally, EIF4A1P5 is an initiation factor in the 43 ribosome that assists with cytoplasmic translation and is found to have oncogenic properties in lung cancers [8]. Of these genes, GAS5-AS1 is an oncogene that would be beneficial for possible knock-out studies to determine its overall effect on lung cancer proliferation

The top three downregulated genes were EIF1B, NAPIL1, and TPT1. EIF1B is a translation regulator that is an oncogene in mixed-cell uveal melanoma [9]. NAPIL1 is a nucleosome assembly protein that is dysregulated in lung and ovarian cancer ([10]. TPT1 is a long non-coding RNA that is dysregulated in prostate cancer [11]. Interestingly we found that four known tumor suppressors exist in the downregulated genes associated with other type of cancers (other than small-cell lung cancer). The tumor suppressors PDCD4-AS1, RNF40, USP4, and ST13P5 are good candidates for knock-in experiments to see if an increase in expression causes less or more proliferation [12].

The small non-coding RNA sequencing data can provide insight into new possible targets for cancer progression and induction. Of the data, most of the genes came in the form of tRNA/tRNA fragments. Transfer RNAs (tRNAs) are non-coding transcripts that introduce amino acids to ribosomes for protein synthesis. tRNA fragments (tRFs) are generated by specific angiogenin (ANG) cleavage under stress conditions and function similarly to miRs [13]. There is increasingly more evidence that these tRNA and tRNA fragments play roles in gene expression therefore, could be used as clinical biomarkers and therapeutic targets. However, more research is still needed to determine both the expression implications and the functions of these tRNA/tRNA fragments in cancer development and progression.

In the small non-coding RNA sequencing data, we found that miR892B that is a known tumor suppressor in breast cancer was downregulated -. Downregulation of miR892b can increase the activation of *nf-kb*, a gene that assists with the proliferation of various cancers [14].

## 5. IPA Analysis of AG12211 Compared to HTB-172 Transcriptomic Data

The most significant finding of the IPA analysis is the differential expression of oncogenes based on RNA sequencing data. A few genes conserved in each of these pathways are possible genes of interest in further studies. For example, the gene that was conserved the most in the pathways, the PIK3R1 gene, was in five of the seven pathways that were being analyzed in the top network. The PIK3R1 gene encodes for a kinase that is found to be dysregulated in ovarian and colon tumors, PIK3R1 is also involved in EMT and proliferation in renal cell carcinoma [15]. The oncogenes PIK3R1, FOXO3, TP53, BAK1, and NF-kappa beta were all found in two or more of the seven overlayed pathways and would serve as potential target genes for further investigation.

The core canonical pathways used to find the oncogenes that were conserved in all of the pathways were EIF2 signaling, VEGF signaling, HOTAIR signaling, IL-13 signaling, and UVB-induced MAPK signaling. Phosphorylation of EIF2 (Eukaryotic Translation Initiation Factor 2), induced by endoplasmic reticulum (ER) stress, activating transcription factor 4 (ATF4), which affects tumor microenvironment immune cell response due to activation of unfolded protein response (UPR). UPR is caused by the accumulation of unfolded proteins in the ER in response to stress. UPR is involved in tumor initiation and progression [16]. The upregulation of these pathways is essential to the Tumor Micro Environment (TME) story because this pathway attributes to immune system evasion and tumor progression.

The second upregulated pathway in this study was the VEGF pathway. VEGF (Vascular and Endothelial Growth Factor) pathway is involved in developing the tumor blood vessels (angiogenesis). The VEGF pathway is activated by hypoxia-induced factor 1a (HIF-1a), deregulation of growth factors, and increased proliferation signals. In addition, the VEGF pathway is involved in immune system evasion by the inhibition of dendritic cells. It is also a key player in TME development because of the increase in nitrogen oxide (NO), a biomarker of TME [17].

The final upregulated pathway identified was the HOTAIR signaling pathway. HOTAIR (HOX Transcript Antisense Intergenic RNA) is a long non-coding RNA transcript associated with NFK-B activity [18]. HOTAIR is a scaffolding between PRC2 and LSD1 (Lysine-specific histone demethylase 1A). LSD1 forms a protein complex with gene-specific repressor element RE-1 silencing transcription factor (REST) and CoREST, critical players in gene silencing. In addition, HOTAIR activates PRC2 methylates chromatin with the help of JARID2 (Jumonji and AT-Rich Interaction Domain Containing 2), and LSD1 demethylates H3 lysine K4 [19]. These interactions between HOTAIR, PRC2, and LSD1 could block the transcription of target genes by interrupting chromatin folding [20]. Herein, we observed the upregulation of this pathway in HTB-172 compared to AG12211 corresponds to the upregulation of the EZH2 by RT-PCR and ELISA analyses. These results suggest an alternative pathway in small-cell lung cancer as opposed to the mir200 pathway that was previously found in the adenocarcinoma A549 cell line [21].

Two downregulated canonical pathways were investigated in this study, and those pathways are IL-13 (Interleukin 13) and UVB-induced MAPK signaling. IL-13 is a cell signaling protein (cytokine) that phosphorylates the transcription factor STAT6 [22]. This pathway has been shown to activate the ZEB1 EMT transcription factor and participates in the activation of tumor-associated macrophages and promote proliferation, survival, and metastasis in colorectal cancer [23]. According to IPA, UVB-induced MAPK signaling is activated by UV radiation which triggers the downregulation of PKC (protein kinase C) and p38 MAPK. Notably, this pathway could decrease chromatin remodeling, cell proliferation, and tumor production [24]. The pattern of activation versus deactivation of the canonical pathways shows that even if a pathway induces or accelerates one cancer, that same pathway can have the opposite effect in others, as is in the case of AG12211 versus HTB-172.

While observing the overlayed pathways with the top network, the gene for ezh2 was seen but was not represented in the HTB-172 versus AG12211 data. However, EZH2 appears to have a relationship with other genes and molecules, including the microRNA gene *malat1* that also had a predicted relationship to vimentin, the inhibition of *cadherin* genes, and the upregulation of TWIST1, which is a transcription factor involved in EMT [25].

Understanding the major functional and transcriptional regulation of cancer is important to provide more insight into the molecular mechanisms regulating cancer cell behavior. There are only few published manuscripts on SCLC [26]-[28]; Taken together, we investigated the role of differentially expressed genes in SCLC, the most common form of lung cancer in smokers worldwide, and reported the core canonical pathways involved in cell signaling of these dreaded lung cancer cells, Further investigations and research targeting the genes involved in the core canonical pathways reported in this manuscript would result in developing targets for prognostic and therapeutic strategies and interventions of this deadly cancer affecting smokers all around the world.

## Figures and Tables

**Table 1.** Raw data of whole transcriptomic RNA sequencing. This data represents the top three genes with the greatest fold change upregulation (red color) of Ag12211 vs HTB-172. GAS5-AS1 has the greatest upregulated fold change and has been established as an oncogene in lung and hepatocellular carcinoma.

Raw Whole Transcriptome RNASeq Data (Top 3 Upregulated Fold Change)					
Gene ID	Gene Name	FC	Location	Type	Association
*GAS5-AS1*	GAS5 antisense RNA 1	+35	Other	lncRNA	Lung Cancer and Hepatocellular Carcinoma
*OR1A2*	olfactory receptor family 1 subfamily A member 2	+25	Plasma Membrane	G-protein coupled receptor	Olfactory Receptor
*EIF4A1P5*	eukaryotic translation initiation factor 4A1 pseudogene 5	+24	Other	other	Cytoplasmic translational initiation, Lung Cancer

**Table 2.** Raw data of whole transcriptomic RNA sequencing. This data represents the top three genes with the greatest fold change downregulation (blue color) of Ag12211 vs HTB-172. All three of these genes have oncogenic properties in other cancers.

Raw Whole Transcriptome RNASeq Data (Top 3 Downregulated Fold Change)					
Gene ID	Gene Name	FC	Location	Type	Association
*EIF1B*	eukaryotic translation initiation factor 1B	−97	Cytoplasm	translation regulator	Mixed Cell Uveal Melanoma
*NAP1L1*	nucleosome assembly protein 1 like 1	−47	Nucleus	nucleosome assembly protein	Oncogene, Lung and Ovarian Cancer
*TPT1*	tumor protein, translationally-controlled 1	−41	Cytoplasm	lncRNA	Oncogene, Pancreatic Cancer

**Table 3.** Raw data of AG12211 compared to HTB-172. Out of top 50 of the 372 downregulated genes, only four were known tumor suppressors.

Raw Whole Transcriptome RNASeq Data (Known Tumor Suppressors)					
Gene ID	Gene Name	FC	Location	Type	Association
*PDCD4-AS1*	PDCD4-Antisense RNA 1	−17	Cytoplasm	lncRNA	Breast Cancer
*RNF40*	Ring Finger Protein 40	−15	Nucleus	E3 ubiquitin-protein ligase	Colorectal cancer
*USP4*	Ubiquitin Specific Peptidase 4	−14	Cytoplasm	protease	Adenocarcinoma and Breast Cancer
*ST13P5*	ST13, Hsp70 interacting protein pseudogene 5	−12	Extracellular	pseudogene	Colorectal cancer

**Table 4.** Raw data of small non-coding RNA sequencing. This data represents the genes that have greater than 10-fold upregulation (red color) in Ag12211 vs HTB-172. Some tRNA or tRNA fragments have been found to play a role in cancer development and proliferation. tRNA/tRNA fragments are currently being investigated for potential treatment targets.

Small Non-Coding RNA Upregulation			
Gene ID	Gene (upregulated)	FC	Association
chr10.trna6	trna6	+29	Unknown
chr2.trna21	trna21	+22	Unknown
chr19.trna13	trna13	+13	Unknown
chr2.trna14	trna14	+11	Unknown

**Table 5.** Raw data of small non-coding RNA sequencing. This data represents the genes that have greater than 10-fold downregulation (blue color) in Ag12211 vs HTB-172. Some tRNA or tRNA fragments have been found to play a role in cancer development and proliferation. tRNA/tRNA fragments are currently being investigated for potential treatment targets. The has-mir-892B gene encodes mir892b and is the only microRNA found in the sequencing. mIR892 has been found to play a role in breast cancer progression.

Small Non-Coding RNA Downregulation			
Gene ID	Gene	FC	Association
chr6.trna108	trna108	−14	Unknown
chr6.trna101	trna101	−10	Leukemia and Lung cancer
chr6.trna102	trna102	−10	Unknown
MI0005538	hsa-mir-892b	−5	Breast Cancer

**Table 6.** Disease and function analysis of upregulated (red color) genes associated with lung cancer proliferation in AG12211 healthy cell line versus small-cell lung cancer line HTB-172.

Upregulated Genes Associated with Lung Cancer Proliferation in AG12211 Compared to HTB-172								
GENE	Entrez Gene Name	Expr Log Ratio	Location	Family	Association	Known Function	Data Set	Predicted Effect On Function
FOXO3	forkhead box O3	+3.8	Nucleus	transcription regulator	Secondary Acute Leukemia	Decrease Proliferation	Upregulated	Decrease Proliferation
TUSC1	tumor suppressor candidate 1	+2.4	Other	other	Duodenal Obstruction and Lung Cancer	Decrease Proliferation	Upregulated	Decrease Proliferation
GAPDH	glyceraldehyde-3-phosphate dehydrogenase	+2.1	Cytoplasm	enzyme	Alzheimer’s and Various Cancers	Increase Proliferation	Upregulated	Increase Proliferation
LOC102724428/SIK1	salt inducible kinase 1	+1.6	Nucleus	kinase	Oncogene	Increase Proliferation	Upregulated	Increase Proliferation
BAK1	BCL2 antagonist/killer 1	+1.5	Cytoplasm	other	Cancer Tumor Suppressor	Decrease Proliferation	Upregulated	Decrease Proliferation
PIK3R1	phosphoinositide-3-kinase regulatory subunit 1	+1.4	Cytoplasm	kinase	Ovarian and colon tumors	Increase Proliferation	Upregulated	Increase Proliferation
ENO1	enolase 1	+1.0	Cytoplasm	enzyme	Oncogene Various Cancers	Increase Proliferation	Upregulated	Increase Proliferation
FZD9	frizzled class receptor 9	+2.4	Plasma Membrane	G-protein coupled receptor	Williams Syndrome	Unknown	Upregulated	Unknown
TP53	tumor protein p53	+1.0	Nucleus	transcription regulator	Tumor Suppressor	Unknown	Upregulated	Unknown

**Table 7.** Disease and function analysis of downregulated genes associated with lung cancer proliferation in AG12211 healthy cell line versus small-cell lung cancer line HTB-172.

Downregulated Genes Associated with Lung Cancer Proliferation in AG12211 Compared to HTB-172								
GENE	Entrez Gene Name	Expr Log Ratio	Location	Family	Association	Known Function	Data Set	Predicted Effect On Function
EEF1A2	eukaryotic translation elongation factor 1 alpha 2	−1.5	Cytoplasm	translation regulator	Ovarian Cancer	Increase Proliferation	Downregulated	Decrease Proliferation
COPB2	COPI coat complex subunit beta 2	−1.5	Cytoplasm	transporter	Oncogene (Breast, *et al)*	Increase Proliferation	Downregulated	Decrease Proliferation
CNOT3	CCR4-NOT transcription complex subunit 3	−2.0	Cytoplasm	other	mRNA degradation, ALL	Increase Proliferation	Downregulated	Decrease Proliferation
PRKCI	protein kinase C iota	−3.5	Cytoplasm	kinase	Glioblastoma and Lung Cancer	Increase Proliferation	Downregulated	Decrease Proliferation

**Table 8.** Disease and function analysis of upregulated (red color) genes associated with apoptosis in lung cancer in AG12211 healthy cell line versus small-cell lung cancer line HTB-172.

Upregulated Genes Associated with Lung Cancer Apoptosis in AG12211 Compared to HTB-172								
GENE	Entrez Gene Name	Expr Log Ratio	Location	Family	Association	Known Function	Data Set	Predicted Effect On Function
FOXO3	forkhead box O3	+3.8	Nucleus	transcription regulator	Secondary Acute Leukemia	Increase Apoptosis	Upregulated	Increase Apoptosis
YBX1	Y-box binding protein 1	+2.4	Nucleus	transcription regulator	Oncogene Various Cancers	Decrease Apoptosis	Upregulated	Decrease Apoptosis
BAK1	BCL2 antagonist/killer 1	+1.5	Cytoplasm	other	Cancer Tumor Suppressor	Increase Apoptosis	Upregulated	Increase Apoptosis
SNRPE	small nuclear ribonucleoprotein polypeptide E	+1.3	Nucleus	other	Lupus Erythematosus, Breast Cancer	Decrease Apoptosis	Upregulated	Decrease Apoptosis
RRM2	ribonucleotide reductase regulatory subunit M2	+1.2	Nucleus	enzyme	Ovarian Cancer, Breast and NSCLC	Decrease Apoptosis	Upregulated	Decrease Apoptosis
ATP1A1	ATPase Na+/K+ transporting subunit alpha 1	+1.0	Plasma Membrane	transporter	EMT of tumor cells and myofibroblast activation	Involved in Apoptosis	Upregulated	Unknown
COX6B1	cytochrome c oxidase subunit 6B1	+1.0	Cytoplasm	enzyme	Mitochondrial Complex Iv Deficiency, Nuclear Type 7	Decrease Apoptosis	Upregulated	Decrease Apoptosis
TP53	tumor protein p53	+1.0	Nucleus	transcription regulator	Tumor Suppressor	Increase Apoptosis	Upregulated	Increase Apoptosis

**Table 9.** Disease and function analysis of downregulated (blue color) genes associated with apoptosis in lung cancer in AG12211 healthy cell line versus small-cell lung cancer line HTB-172.

Downregulated Genes Associated with Lung Cancer Apoptosis in AG12211 Compared to HTB-172								
GENE	Entrez Gene Name	Expr Log Ratio	Location	Family	Association	Known Function	Data Set	Predicted Effect On Function
EEF1A2	eukaryotic translation elongation factor 1 alpha 2	−1.5	Cytoplasm	translation regulator	Oncogene, Ovarian cancer	Decrease Apoptosis	Downregulated	Increase Apoptosis
NFKBIA	NFKB inhibitor alpha	−1.5	Cytoplasm	transcription regulator	Ectodermal Dysplasia And Immunodeficiency 2	Decrease Apoptosis	Downregulated	Increase Apoptosis
VDAC1	voltage dependent anion channel 1	−1.5	Cytoplasm	ion channel	Mesothelioma and Breast Cancer	Involved in Apoptosis	Downregulated	Unknown
